# Prevalence of antiphospholipid antibodies in Behçet's disease: A systematic review and meta-analysis

**DOI:** 10.1371/journal.pone.0227836

**Published:** 2020-01-13

**Authors:** Md Asiful Islam, Sayeda Sadia Alam, Shoumik Kundu, A. H. M. Safayet Ullah Prodhan, Shahad Saif Khandker, Tatiana Reshetnyak, Przemysław J. Kotyla, Rosline Hassan, Tareq Hossan

**Affiliations:** 1 Department of Haematology, School of Medical Sciences, Universiti Sains Malaysia, Kubang Kerian, Kelantan, Malaysia; 2 Department of Biochemistry and Molecular Biology, Jahangirnagar University, Savar, Dhaka , Bangladesh; 3 Department of Systemic Rheumatic Disease, V.A. Nasonova Research Institute of Rheumatology, Moscow, Russia; 4 Department of Internal Medicine, Rheumatology and Clinical Immunology, Faculty in Katowice, Medical University of Silesia, Katowice, Poland; King Abdulaziz University, SAUDI ARABIA

## Abstract

Behçet’s disease (BD) is a multifactorial systemic inflammatory disease of unknown aetiology characterised by several clinical manifestations including vascular involvements (*i*.*e*., both arterial and venous thrombosis). Antiphospholipid antibodies (aPLs)—including anticardiolipin (aCL), anti-β2-glycoprotein I (β2-GPI) antibodies and lupus anticoagulant (LA) are detected in systemic autoimmune diseases which contribute to thrombosis. The aim of this systematic review and meta-analysis was to evaluate the prevalence of aPLs in patients with BD as compared to controls. A protocol was registered in PROSPERO (Registration No. CRD42018088125) and a systematic literature search was conducted through PubMed, Web of Science, Embase, Scopus and ScienceDirect databases. Odds ratios (ORs) and 95% confidence intervals (CIs) were calculated using random-effects model. Quality assessment was carried out by using the modified 9-star Newcastle-Ottawa Scale (NOS). Publication bias was evaluated via visualisation of contour- enhanced and trim and fill funnel plots along with Begg's and Egger's tests. We included ten case-control studies (a total of 999 participants from 380 BD patients and 619 controls) based on the inclusion criteria. The prevalence of aCL (OR: 12.10, 95% CI: 5.15–28.41, *p*<0.00001) and anti-β2-GPI antibodies (OR: 23.57, 95% CI: 1.31–423.63, *p* = 0.03) were statistically significant, however, the prevalence of LA was not significant (OR: 13.77, 95% CI: 0.65–293.59, *p* = 0.09). The results remained statistically significant from different sensitivity analyses which represented the robustness of this meta-analysis. According to the NOS, 50.0% of the studies were considered as of high methodological quality (low risk of bias). No significant publication bias was detected from contour-enhanced and trim and fill funnel plots or Begg's and Egger’s tests. This meta-analysis established that there is a significantly high prevalence of aPLs (*i*.*e*., aCL and anti-β2-GPI antibodies) in patients with BD when compared to controls.

## Introduction

Behçet’s disease (BD) is a systemic inflammatory disease with a chronic, relapsing-remitting course of unknown aetiology, however, presumed to be multifactorial, implicating genetic, infectious and immunologic factors. The disease is characterised by a range of clinical manifestations including recurrent oral and genital ulcers, skin lesions, ocular, vascular, articular, gastrointestinal, urogenital, pulmonary and neurologic involvements. In absence of universally accepted diagnostic laboratory test, the diagnosis can only be made based on clinical symptoms and signs [1–3]. Patients with BD share some common features with autoimmune and autoinflammatory diseases [4–8]. The prevalence of BD is higher in the ancient silk route areas—geographically the Mediterranean and the Middle East regions (between the latitudes of 30°N and 45°N) [9, 10] with an estimated worldwide prevalence of 10.3/100,000 and is equally distributed in men and women [11, 12].

Vascular events affect up to 45% of BD patients involving both arterial and venous vessels of all sizes, but deep vein thrombosis (DVT) and superficial vein thrombosis (SVT) of the lower extremities are the most common vascular manifestations of the disease [13, 14]. Systemic inflammation, more than usual thrombophilic factors is thought to be the main trigger of thrombosis in this condition and seems to be mainly mediated by T-cells, monocytes, neutrophils and proinflammatory cytokines along with endothelial cell dysfunction [15, 16]. A group of autoantibodies generally referred to as antiphospholipid antibodies (aPLs)—which include anticardiolipin (aCL) antibodies, anti-β2-glycoprotein I (β2-GPI) antibodies and lupus anticoagulant (LA) have been observed in autoimmune diseases including BD [17–20]. The combination of these autoantibodies and their level is important in assessing the risk of thrombosis [21]. Presence of aPLs has been detected in patients with BD [22–24], however, inconclusive. Since recurrent thrombosis, thrombocytopenia and frequent vasculitis are common manifestations of BD, there is a need to investigate aPLs in serum of these patients [25, 26].

To the best of our knowledge, there is no systematic review and meta-analysis assessing the presence of aPLs in patients with BD. Therefore, the aim of this systematic review and meta-analysis was to assess the prevalence of aPLs (*i*.*e*., aCL, anti-β2-GPI and LA) in patients with BD as compared to controls.

## Methods

### Eligibility criteria

This systemic review and meta-analysis was developed in accordance with the guidelines and recommendations of *Meta-analysis of Observational Studies in Epidemiology* (MOOSE) [27] (S1 Table) and *Preferred Reporting Items for Systematic Review and Meta-Analysis* (PRISMA) Statements [28] (S2 Table). A predefined protocol was registered with PROSPERO (an international database of prospectively registered systematic reviews), University of York, York, UK (Registration No. CRD42018088125). Case-control studies assessing the presence or absence of aPLs [LA, aCL, anti-β2-GPI, antiprothrombin (aPT), antiphosphatidylserine (aPS), antiphosphatidylinositol (aPI) and antiphosphatidylethanolamine (aPE) antibodies] in BD [without any underlying autoimmune diseases including antiphospholipid syndrome (APS) and systemic lupus erythematosus (SLE)] of any age, sex or race were considered eligible patients. Subjects without the history of thrombosis and BD of any age, sex or race were considered eligible control participants.

### Literature search

Search strategies for different databases were developed and comprehensive searches combining the appropriate keywords with Boolean logical operators (‘AND’ & ‘OR’) using ‘Advanced’ and ‘Expert’ search options were conducted. Electronic databases including PubMed, Web of Science, Embase, Scopus and ScienceDirect were searched independently by three authors (MAI, SK and TH) and screened by another three authors (SSA, AHMSUP and SSK). The final systematic search was conducted on May 21, 2019. There were no year and language restrictions. Non-human subjects, review articles, case reports, clinical trials, editorials, letters, comments and duplicate articles among different databases were excluded. Duplicate studies which may result from different electronic databases were removed and managed by EndNote software (version X8). In addition, references in the primary selected studies were also examined to identify any other possible relevant studies.

### Data extraction

**Fig 1:**
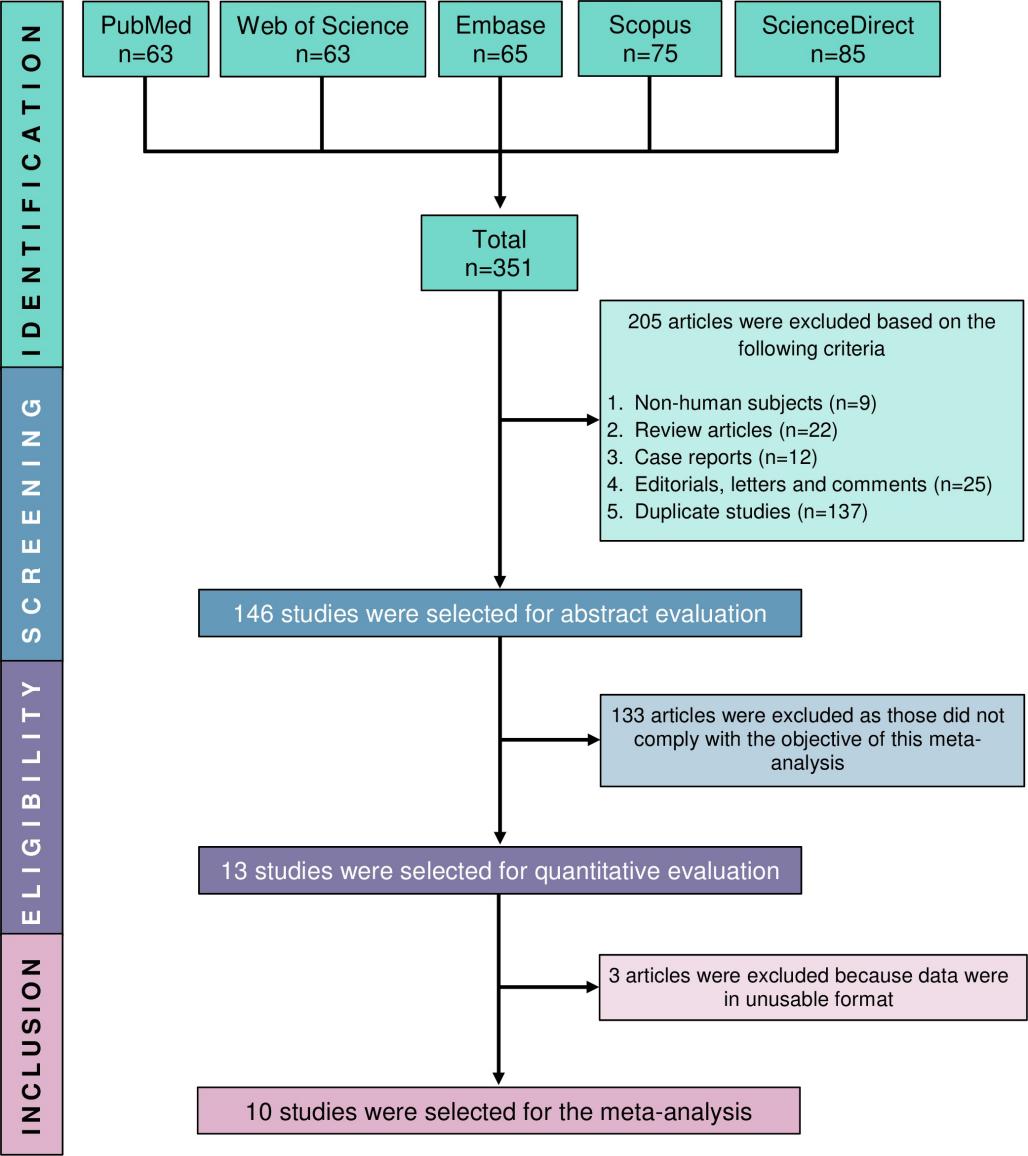
PRISMA flow diagram of study selection.

The studies were selected based on the inclusion criteria and selection methodology as illustrated in Fig 1. The types of data extracted from the selected studies are as follows: study design, country of origin of the participants, age category [adult (age ≥18 years) or paediatric (age <18 years)], number of BD and control subjects, number of male and female subjects of patients and controls, disease duration, types of control subjects, mean/median age of the patients and controls, types and isotypes of tested aPLs, cut-off values and the quantitative data of the presence of aPLs in both patients and controls. Data extraction was done by five authors (MAI, SSA, SK, AHMSUP and TH) and these authors took part in the discussions to resolve any discrepancies, unclear or missing data presentation. If unresolved, either the corresponding or the first author of the respective study was contacted for further clarifications.

### Data analyses

Odds ratio (OR) was used to evaluate the presence of aPLs in BD patients compared to controls, where, *p*<0.05 was considered as statistically significant. Random-effects model was used for the analyses. To assess the heterogeneity (*I*^*2*^) of the included studies, Tau-squared test was used where *I*^*2*^ assessed the quantity of inconsistency across the studies (*p*<0.10 was considered as significant). A value of *I*^*2*^ close to zero indicates homogeneity, whereas, the following ranges of *I*^*2*^ were used to interpret heterogeneity: low heterogeneity if *I*^*2*^ = 25–50%, moderate heterogeneity if *I*^*2*^ = 51–75% and substantial heterogeneity if *I*^*2*^>75% [17]. For visual inspection of heterogeneity, L'Abbé plot was generated. Predefined subgroup analysis was planned to conduct on BD patients from different continents.

Quality assessment of each of the included studies was evaluated by MAI, SSA, SK, AHMSUP and TH based on a modified version (nine-star scoring system) of the Newcastle-Ottawa Scale (NOS) for case-control studies [18]. Studies with NOS scores of above or equal to the median were considered as high quality (low risk of bias) [17, 18, 29]. To visually inspect asymmetry due to publication bias, a contour-enhanced funnel plot was constructed. Additionally, Begg’s and Egger’s tests were performed for the quantitative analysis of publication bias, where, *p*<0.05 was considered as statistically significant. Results of publication bias were further validated by constructing trim and fill funnel plot.

To identify the source of heterogeneity and to check the robustness of the results, sensitivity analyses were performed. Firstly, it was performed by the leave-one-out method (removing one study each time and repeating the analysis). This analysis allowed us to determine the impact of each study on the overall effect size. Secondly, by following the exclusion of studies with NOS score less than the median (poor-quality studies). Thirdly, by changing the analysis method from random-effects model to fixed-effects model [30]. Fourthly, by only considering the studies where the study sample size is ≥100. Additionally, to identify possible sources of heterogeneity, a Galbraith plot was generated.

RevMan (version 5.3.5) [31] was used to generate the forest plots. Begg’s and Egger’s tests, contour-enhanced funnel, trim and fill funnel, L'Abbé plot and Galbraith plots were constructed by using metafor package (version 2.1–0) of R (version 3.6.1) in RStudio (version 1.2.5001) software [32].

## Results

### Selection of studies

Our search strategy (S3 Table) initially yielded a total of 351 articles from PubMed, Web of Science, Embase, Scopus and ScienceDirect databases. After excluding 205 articles in the identification phase (non-human subjects, n = 9; review articles, n = 22; case reports, n = 12; editorial, letter and comments, n = 25 and duplicate studies, n = 137), abstracts of the remaining 146 articles were further screened. A total of 13 studies were considered as eligible, however, three studies were excluded due to unusable data format. Therefore, finally, based on the study objectives and inclusion/exclusion criteria, ten studies [22–24, 33–39] (a total of 999 participants from 380 BD patients and 619 controls) were included in this meta-analysis (Fig 1) and full texts of all the included ten studies were retrieved.

### Study characteristics

The major characteristics of the included studies are presented in Table 1. All of the included case-control studies were journal articles and on adult subjects [22–24, 33–39]. Among these ten studies, five were conducted on European populations across five different countries (Serbia [39], Turkey [23], Spain [37], the UK [35] and Israel [34]), while the remaining five studies were from Asia (Iran [24], Oman [36] and Korea [22, 38]) and Africa (Egypt [33]). All of the ten studies assessed the prevalence of aCL [22–24, 33–39], while anti-β2-GPI antibody was screened in one study [36] and LA in a single study [33]. Different cut-off values were used to confirm aCL antibody-positivity.

**Table 1:** Major characteristics of the included studies in this meta-analysis.

No.	Study ID	Country	Types of subjects	Number of Behçet's disease patients (number of female)	Mean age/range of Behçet's disease patients (years)	Disease duration	Number of controls (number of female)	Control type	Mean age/range of control (years)	Types of tested aPLs (isotype); cut-offs
1	Zivkovic 2011	Serbia	Adult	11 (3)	34.6	NR	11 (NR)	Healthy	NR	aCL (IgG, IgM, IgA); ≥ 10.0 GPL / MPL
2	EL-Nakeeb 2006	Egypt	Adult	25 (12)	36.8 ± 5.7	NR	25 (17)	Subjects with rheumatic diseases without thrombosis; osteoarthritis (n = 10), rheumatoid arthritis (n = 9), psoriatic arthritis, (n = 3) and fibromyalgia (n = 3)	39.0 ± 4.2	aCL (IgG, IgM, IgA); NR, LA
3	Musabak 2005	Turkey	Adult	33 (5)	21–52	0–28	20 (3)	Healthy	21–48	aCL (IgG, IgM); ≥ 12 GPL and ≥ 13.0 MPL
4	Rajaee 2004	Iran	Adult	G1: 40 (29) G2: 40 (24)	G1: 36.12 ± 7.83 G2: 30.65 ± 7.91	G1: 1–25 G2: 1–11	80 (NR)	Healthy	NR	aCL (IgG); ≥ 10.0 GPL
5	Espinosa 2002	Spain	Adult	38 (17)	27.0 ± 12.0	NR	100 (54)	Healthy	41.0 ± 18.0	aCL (IgG, IgM); NR, LA
6	El-Ageb 2002	Oman	Adult	34 (18)	32.8 ± 9.8	6.4 ± 3.2	27 (13)	Healthy	26.2 ± 8.3	aCL (IgG, IgM); ≥ 15.0 GPL / 12.5 MPL; anti-β2-GPI
7	Kang 1998	Korea	Adult	47 (26)	21–61	1–20	20 (NR)	Healthy	NR	aCL (IgG, IgM); ≥ 10 GPL / MPL
8	Hughes 1998	UK	Adult	18 (NR)	NR	NR	116 (NR)	Healthy	NR	aCL (IgG, IgM, IgA); NR
9	Ji 1992	Korea	Adult	68 (38)	NR	NR	20 (NR)	Healthy	NR	aCL (IgG, IgM); NR
10	Bergman 1990	Israel	Adult	26 (6)	20–68	1–28	200 (NR)	Healthy	NR	aCL (IgG, IgM); NR

aCL: anticardiolipin; anti-β2-GPI: anti-β2-glycoprotein I; LA: lupus anticoagulant; aPT: antiprothrombin; aPI: antiphosphatidylinositol; aPC: antiphosphatidylcholine; aPS: antiphosphatidylserine; aPC: antiphosphatidylcholine; aPE: antiphosphatidylethanolamine; KCT: kaolin clotting time; OD: optical density; SD: standard deviation; NR: not reported.

### Prevalence of aCL in Behçet's disease

**Fig 2:**
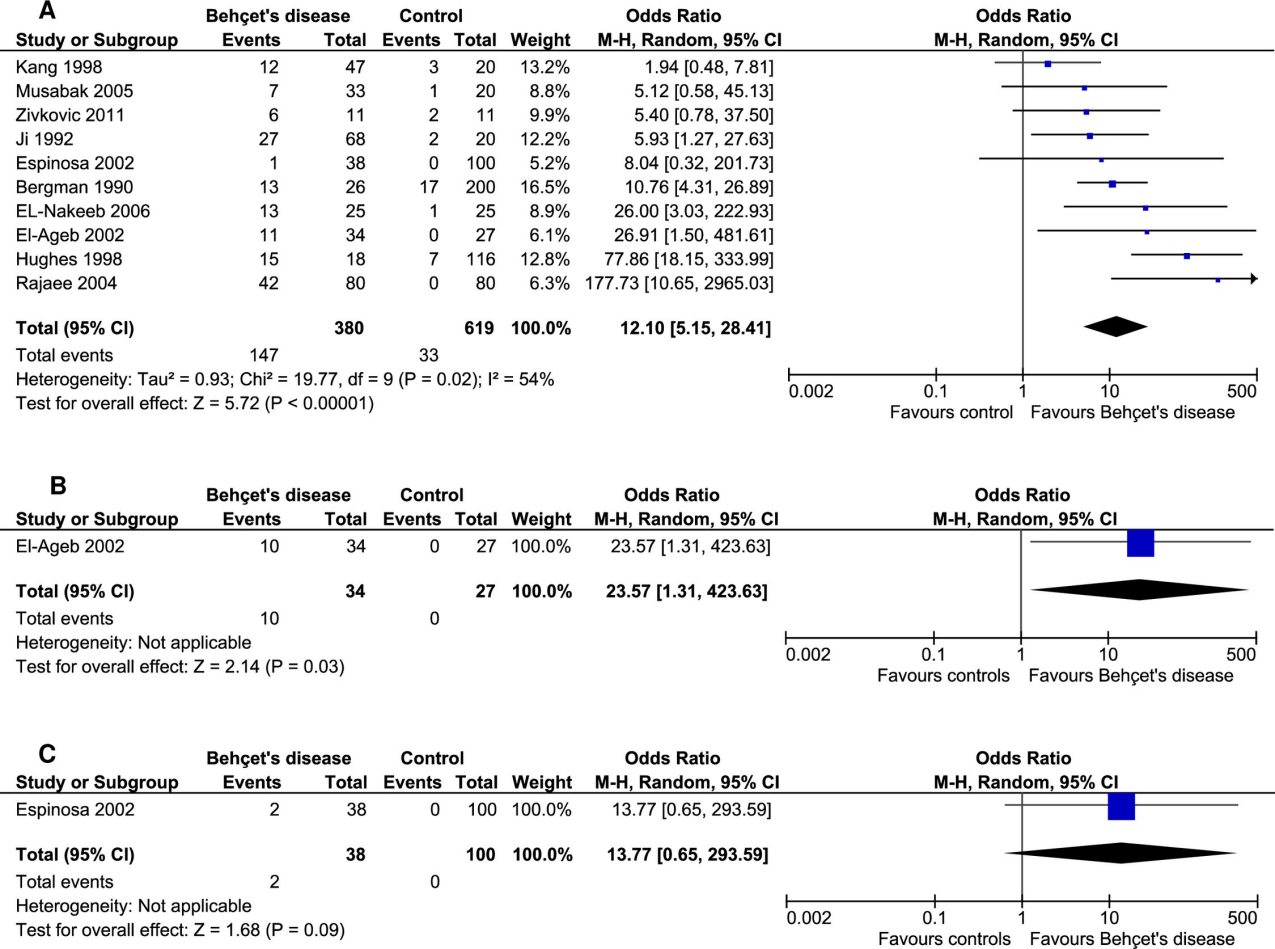
Forest plots showing the prevalence of aCL (A), anti-β2-GPI (B) and LA (C) in Behçet's disease compared to controls.

All of the included studies evaluated the presence of aCL which was reported in 37.11% of the BD patients and 5.33% in controls. The prevalence of aCL antibodies was statistically significant in patients with BD as compared to controls (OR: 12.10, 95% CI: 5.15–28.41, *p*<0.00001; *I*^*2*^ = 54%, *p* = 0.02) (Fig 2A).

### Prevalence of anti-β2-GPI and LA in Behçet's disease

The prevalence of anti-β2-GPI antibodies was estimated in a single study [36], where it was positive in 29.41% of the BD patients and 0.0% of the controls. The prevalence of anti-β2-GPI antibodies was significant in BD patients compared to controls (OR: 23.57, 95% CI: 1.31–423.63, *p* = 0.03) (Fig 2B). On the other hand, only one study assessed the prevalence of LA [37] where it was positive in two BD patients but none was found positive in controls (OR: 13.77, 95% CI: 0.65–293.59, *p* = 0.09) (Fig 2C).

### Subgroup analyses of studies from Europe, Asia and Africa

**Fig 3:**
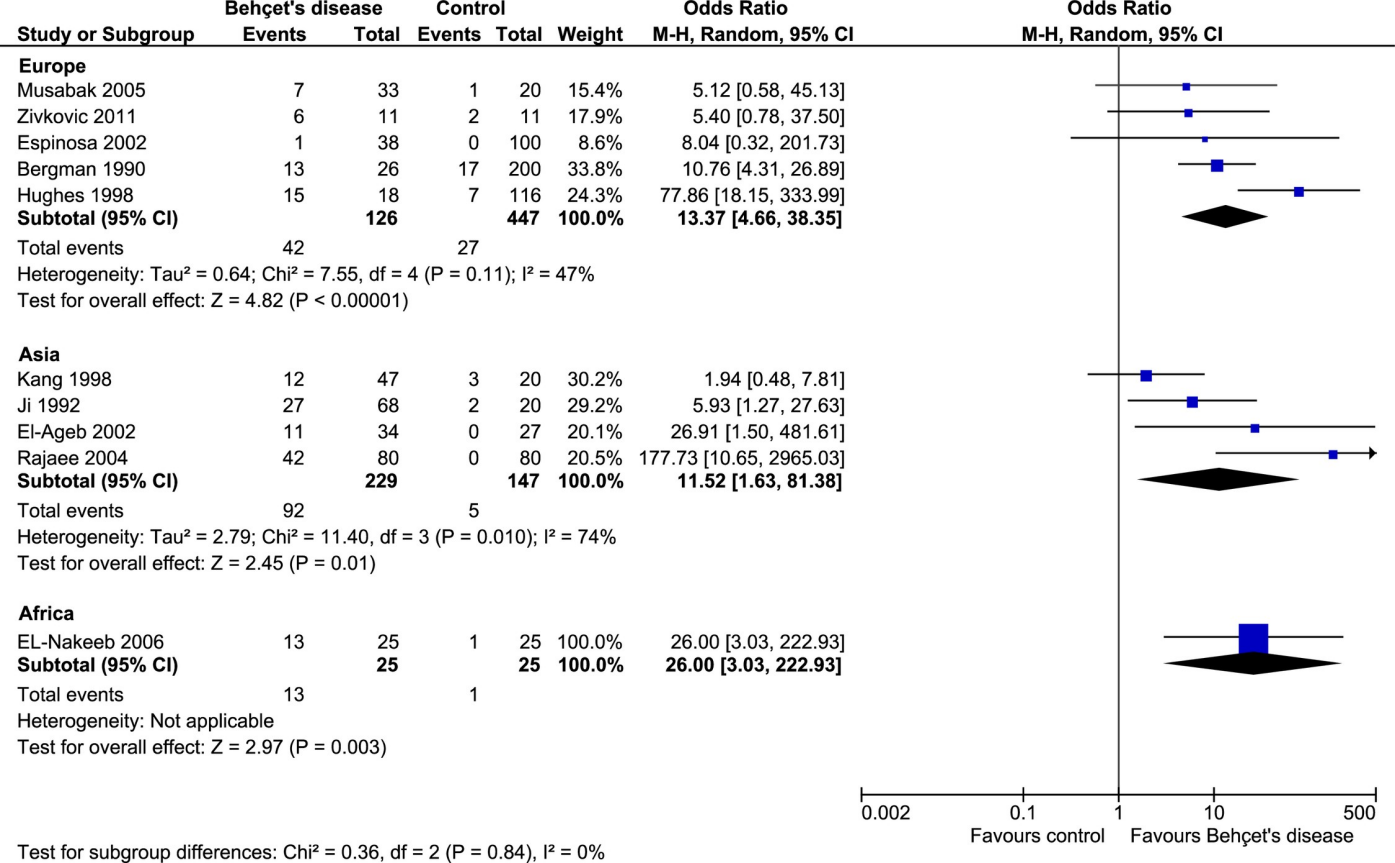
Subgroup analysis on the prevalence of aCL antibodies in European, Asian and African patients with Behçet's disease.

The prevalence of aCL was significant in BD subjects of Europe (OR: 13.37, 95% CI: 4.66–38.35, *p*<0.00001; *I*^*2*^ = 47%, *p* = 0.11), Asia (OR: 11.52, 95% CI: 1.63–81.38, *p* = 0.01; *I*^*2*^ = 74%, *p* = 0.01) and Africa (OR: 26.00, 95% CI: 3.03–222.93, *p* = 0.003) when compared to controls (Fig 3).

### Heterogeneity and quality assessments

**Fig 4:**
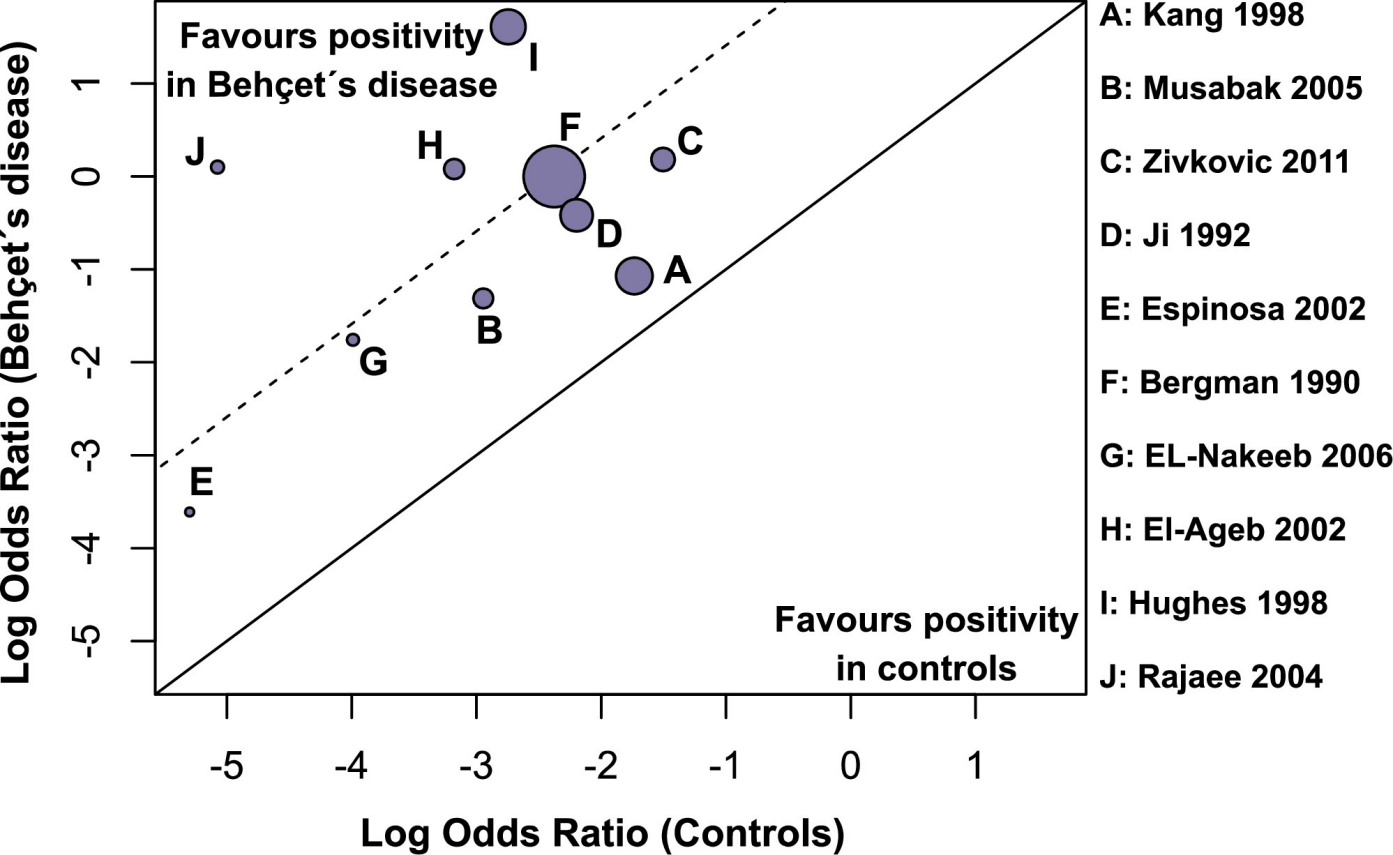
L'Abbé plot for the prevalence of aCL antibodies in Behçet's disease and controls suggests no substantial heterogeneity.

Moderate level of heterogeneity was observed from the forest plot assessing aCL antibodies (*I*^*2*^ = 54%). Additionally, visual evaluation of the L'Abbé plot demonstrated that there was no substantial heterogeneity in the studies assessing aCL antibodies (Fig 4). Quality assessment of the included studies by using NOS for case-control studies is presented in Table 2. The median score of NOS was 7. Therefore, among the ten studies, five were considered as of high methodological quality (low risk of bias) which scored ≥7 [23, 33, 36–38], whereas, the other five studies [22, 24, 34, 35, 39] which scored <7 were therefore considered as of low methodological quality studies (high risk of bias).

**Table 2:** Risk of bias assessment of the included studies according to the modified Newcastle-Ottawa Scale (NOS).

NOS items / Study ID	Zivkovic 2011	EL-Nakeeb 2006	Musabak 2005	Rajaee 2004	El-Ageb 2002	Espinosa 2002	Hughes 1998	Kang 1998	Ji 1992	Bergman 1990
**Is the case definition adequate?**	★	★	★	★	★	★	●	★	★	★
**Representativeness of the cases**	●	★	★	●	★	★	★	●	★	●
**Selection of controls**	★	●	★	★	★	★	★	★	★	★
**Definition of controls**	★	★	★	★	★	★	★	★	★	★
**Study controls for the most important factor (*i*.*e*., age)**	●	★	★	●	●	●	●	●	●	●
**Study controls for the second important factor (*i*.*e*., sex)**	●	★	★	●	★	★	●	●	●	●
**Was the measurement method of aPLs described?**	★	★	★	★	★	★	★	★	★	★
**Were the methods of measurements similar for cases and controls (*i*.*e*., ELISA)?**	★	★	★	★	★	★	★	★	★	★
**Non-response rate**	★	★	★	★	★	★	★	★	★	★
**Total Score**	**6**	**8**	**9**	**6**	**8**	**8**	**6**	**6**	**7**	**6**

★ was awarded when the respective information was available.

● was awarded if the respective information was unavailable.

### Sensitivity analyses

**Fig 5:**
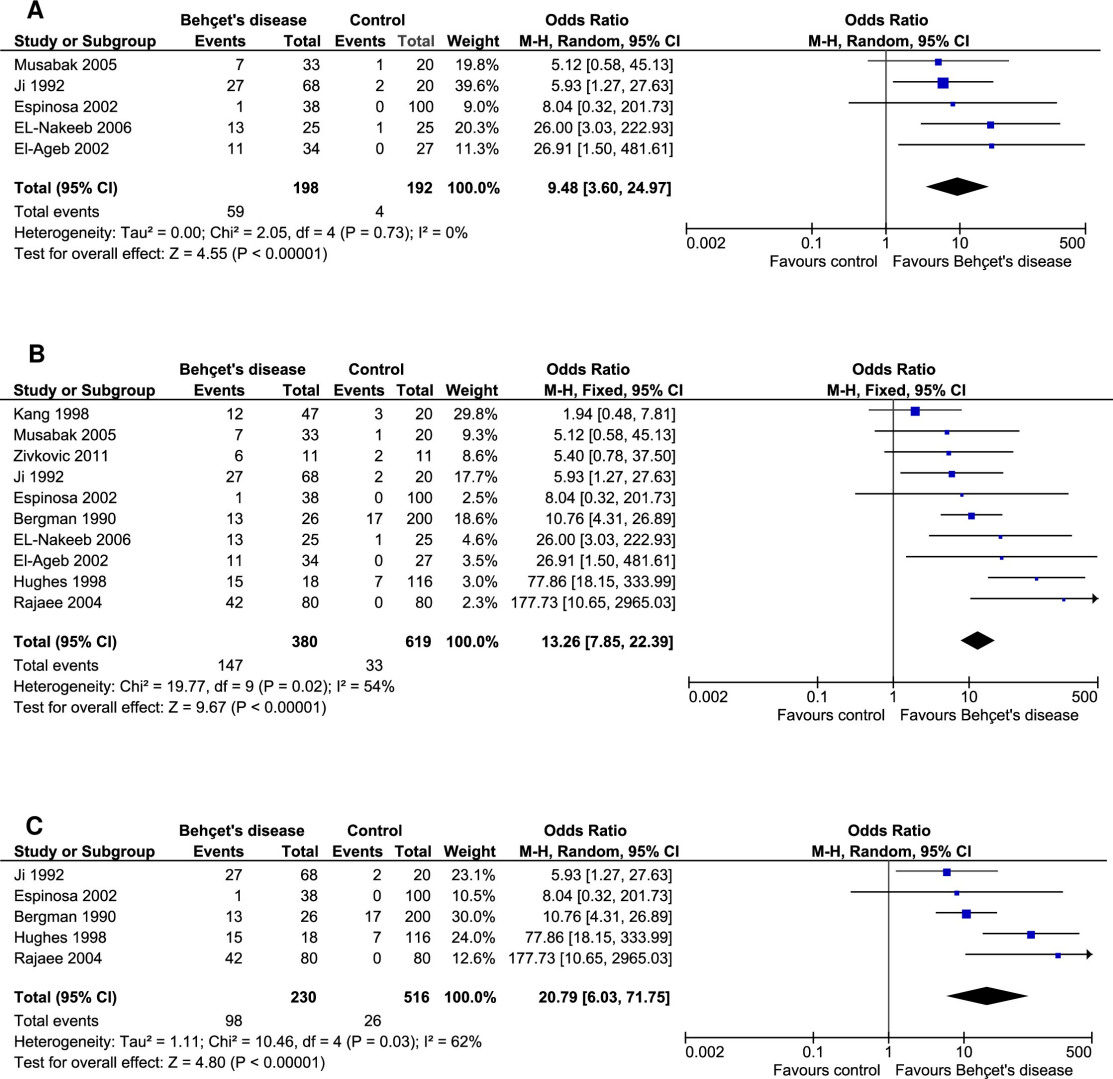
Sensitivity analyses considering A) high-quality studies, B) fixed-effects model, and C) studies with larger sample size (≥100) demonstrated that results remained statistically significant.

Sensitivity analyses revealed that firstly, by excluding individual studies the results were not modified when compared to the main results. Secondly, after excluding the poor-quality studies, the results remained statistically significant (OR: 9.48, 95% CI: 3.60–24.97, *p*<0.00001) (Fig 5A). A third sensitivity analysis with fixed-effects model demonstrated that the results were still statistically significant (OR: 13.26, 95% CI: 7.85–22.39, *p*<0.00001) (Fig 5B). Fourth, while only larger sample size (≥100) studies were considered, the results were persistently significant (OR: 20.79, 95% CI: 6.03–71.75, *p*<0.00001) likewise the main findings (Fig 5C). Therefore, overall, sensitivity analyses revealed that the results produced in this meta-analysis were robust.

### Publication bias assessment

**Fig 6:**
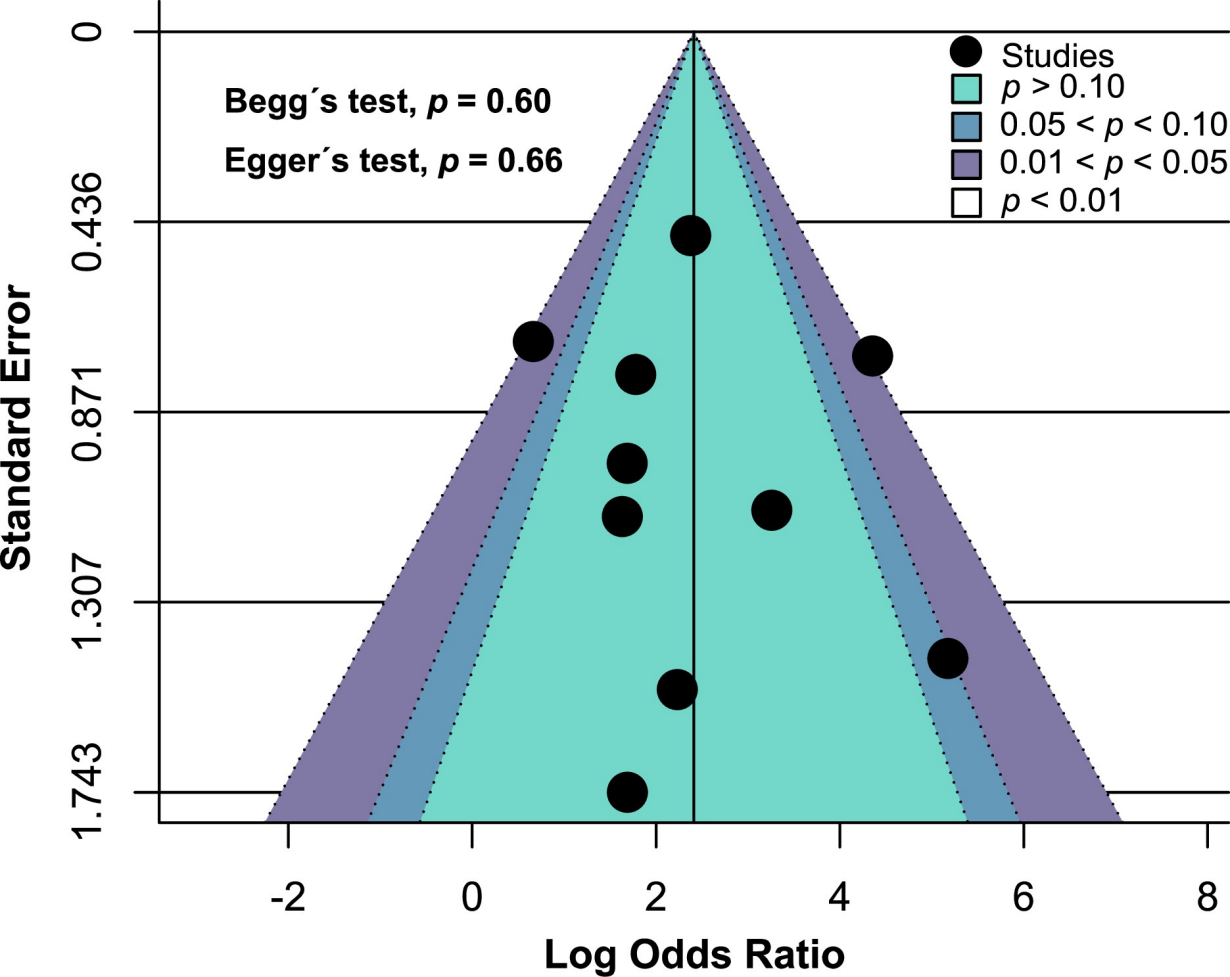
Contour-enhanced funnel plot of the included studies assessing aCL antibodies shows no evidence of publication bias.

Contour-enhanced funnel plot representing the prevalence of aCL antibodies in patients with BD (Fig 6) was used to evaluate publication bias in this meta-analysis. Based on visual inspection of the contour-enhanced funnel plot, it seems that there is no potential publication bias in the studies assessing aCL antibodies. This qualitative result was quantitatively validated and neither Begg’s (*p* = 0.60) nor Egger’s (*p* = 0.66) tests were statistically significant representing no evidence publication bias. Further, publication bias was evaluated by trim and fill funnel plot and there was no evidence of missing studies suggesting no potential publication bias (S1 Fig). Possible contributor of heterogeneity was not identifiable even after constructing the Galbraith plot (S2 Fig).

## Discussion

The results of this meta-analysis indicate towards a significant presence of aPL antibodies (*i*.*e*., aCL and anti-β2-GPI) in BD patients compared to controls.

It has been established that the presence of aPL antibodies especially LA, aCL and anti-β2-GPI can contribute to thrombotic complications [40]. Thrombosis is not a rare clinical event observed in patients with BD. Erol *et al* [41] observed that 77.4% of the Turkish BD patients experienced DVT and 45.2% exhibited pulmonary embolism. In a cohort of Tunisian patients, 30% of them were with recurrent DVT, where, DVT presented in 15% of the BD patients as the first clinical sign [42]. In a group of Iranian patients with BD, elevated levels of aCL antibodies were observed in 50% of the patients with thrombosis [24]. Musabak *et al* [23] postulated aCL antibodies as one of the risk factors in developing atherothrombosis in patients with active BD. Statistically significant association of retinal vascular pathology, cerebral infarction or thrombophlebitis and presence of aCL antibodies were also observed [43]. Most thrombotic events in BD is believed to be attributed through the endothelial injury secondary to vasculitis [44, 45], however, in BD, the mechanisms by which endothelial cell injuries activate the coagulation pathways are largely unknown. It is important in determining the role of the presence of aPLs for therapy of BD patients. Due to primary inflammatory vascular origin, the thrombotic events in BD are better responsive to glucocorticoids or immunosuppressive drugs than to anticoagulants. While standard APS therapies include long-term (almost lifelong) use of anticoagulants. It should be noted that the inappropriate use of anticoagulants in BD could be very dangerous especially in patients with pulmonary artery aneurisms because of the risk of vascular rupture.

Arterial aneurysm is one of the most common vascular complications in BD the prevalence of which is about 25% [37, 46, 47]. Interestingly, aneurysms have been observed in patients with aPLs [48, 49]. At the same time, patients with arterial aneurysm may have aPLs as an additional risk factor for thrombosis. Perhaps the persistent positivity of autoantibodies after a decrease in activity will be an indication for anticoagulant therapy in future.

Neuro-BD represents an important differential diagnosis for primary APS with predominant cerebral vessels involvement. Cerebral venous sinus thrombosis and parenchymal lesions present in both diseases. Significantly high-levels of aCL antibodies were detected in different neurological complications including headache, migraine, dementia, epilepsy and cognitive impairment [17–19, 50, 51]. Some studies observed increased levels of aPLs due to long-term treatment with infliximab [anti-tumour necrosis factor (TNF) monoclonal antibody] and corticosteroids [52, 53] which could also be the trigger initiators for aPLs-contribution. Although the literature data revealed a high frequency of aPLs, their significance in BD remains debatable. In most cases it was at low levels. This mainly concerns the determination of antibody levels for positivity. According to the APS diagnostic criteria (2006) to definite new cut-off levels for aCL are >40 GPL or MPL or 99th percentile. It was decided to use a threshold of >99th percentile of controls for both IgG and IgM class anti-β2-GPI to define a positive result [54, 55].

There are some limitations that can be addressed in this systematic review and meta-analysis. First, a relatively small number of studies (n = 10) with relatively small sample size (n = 999) were eligible to be included in this study. Second, since the included study population of our meta-analysis was from Europe (n = 5), Asia (n = 4) and Africa (n = 1) and there were no studies from North America, Latin America and Australia; therefore, the projection of the results may not represent the global scenario. Third, there were differences in the detection, measurement methods and cut-off values of aCL and anti-β2-GPI antibody-positivity among the included studies. Fourth, among the included studies, LA and anti-β2-GPI antibody were assessed in only single study each, therefore, overall, these results represent more towards the prevalence of aCLs. Fifth, according to the NOS, 50% of the studies were of low methodological qualities, therefore, low-quality studies have generated high-risk of bias in our meta-analysis.

Nevertheless, there are several notable strengths in this systematic review and meta-analysis. First, to the best of our knowledge, this systematic review and meta-analysis is the first of its kind to assess the presence of aPLs in patients with BD as compared to controls. Second, a comprehensive and robust literature search without year and language restriction were conducted across four electronic databases following a standard methodology. Third, despite of performing four different sensitivity analyses, the statistical results remained identical which represented the robustness of the findings of this meta-analysis. Fourth, there was no substantial heterogeneity in the analysis. Fifth, no publication bias was observed from both visual (contour-enhanced and trim and fill funnel plots) and quantitative analyses (Begg's and Egger's tests).

## Conclusion

Our meta-analysis established that there is a significantly high prevalence of aPLs (*i*.*e*., aCL and anti-β2-GPI antibodies) in patients with BD when compared to controls.

## Supporting information

S1 Table MOOSE checklist. (DOCX)

S2 Table PRISMA checklist. (DOCX)

S3 Table Search strategies employed for PubMed, Web of Science, Embase, Scopus and ScienceDirect electronic databases. (DOCX)

S1 Fig Trim and fill funnel plot showing no evidence of missing studies suggesting no potential publication bias. (DOCX)

S2 Fig Galbraith plot representing the possible sources of heterogeneity. Studies within the limits are interpreted as homogeneous.
(DOCX)
