# Supplementary material for: Prevalence of antiphospholipid antibodies in Behçet's disease: A systematic review and meta-analysis
Source: PLoS One. 2020 Jan 13;15(1):e0227836. doi: 10.1371/journal.pone.0227836 (PMC6957187; doi:10.1371/journal.pone.0227836)
Supplement: S1 Table — (DOCX) [file pone.0227836.s001.docx]

| *S1 Table.* *MOOSE Checklist* | | |
| --- | --- | --- |
| **Item No** | **Criteria** | **Sentences mentioned in the meta-analysis or appropriate explanations** |
| 1 | Problem definition | A group of autoantibodies generally referred to as antiphospholipid antibodies (aPLs) - which include anticardiolipin (aCL) antibodies, anti-β2-glycoprotein I (β2-GPI) antibodies and lupus anticoagulant (LA) have been observed in autoimmune and neurological diseases.^14-16^ The presence of these antibodies in the serum is the characteristic feature of a kind of systemic autoimmune disease - antiphospholipid syndrome (APS), where, thrombosis (arterial, venous or microvascular) and obstetrical morbidity are the major clinical manifestations.^17^ The combination of antibodies and their level is important in assessing the risk of thrombosis and triple positivity of aPLs with high levels are high risk for thrombosis.^18^ According to most of the case control studies, a higher percentage of thrombosis was found among patients with BD than the controls. |
| 2 | Hypothesis statement | Presence of aPLs has been detected in patients with BD,^25-27^ however, inconclusive. |
| 3 | Description of study outcome(s) | To assess the prevalence of aPLs in patients with BD as compared to controls. |
| 4 | Type of exposure or intervention used | aPLs (aCL, anti-β2-GPI and LA). |
| 5 | Type of study designs used | Case-control studies. |
| 6 | Study population | BD patients without any underlying autoimmune diseases including APS and SLE of any age, sex or race were considered eligible patients. Subjects without the history of thrombosis and BD of any age, sex or race were considered eligible control participants. |
| 7 | Qualifications of searchers (e.g., librarians and investigators) | MAI (PhD), SSA (BSc), SK (BSc), AHMSUP (BSc), SSK (MSc), TR (MD, DSc), PJK (MD, PhD), RH (MMedPath) and TH (PhD). |
| 8 | Search strategy, including time period included in the synthesis and keywords | Search strategies for different databases were developed and comprehensive searches combining the appropriate keywords with Boolean logical operators (‘AND’ & ‘OR’) using ‘Advanced’ and ‘Expert’ search options were conducted. Electronic databases including PubMed, Web of Science, Embase, Scopus and ScienceDirect were searched independently by three authors (MAI, SK and TH) and screened by another three authors (SSA, AHMSUP and SSK). The final systematic search was conducted on May 21, 2019. There were no year and language restrictions. |
| 9 | Effort to include all available studies, including contact with authors | Five authors (MAI, SSA, SK, AHMSUP and TH) took part in the discussions to resolve any discrepancies, unclear or missing data presentation. If unresolved, either the corresponding or the first author of the respective study was contacted for further clarifications. |
| 10 | Databases and registries searched | PubMed, Web of Science, Embase, Scopus and ScienceDirect. |
| 11 | Search software used, name and version, including special features used (*e.g.*, explosion) | Duplicate studies which may result from different electronic databases were removed and managed by EndNote software (version X8). |
| 12 | Use of hand searching (*e.g.*, reference lists of obtained articles) | In addition, references in the primary selected studies were also examined to identify any other possible relevant studies. |
| 13 | List of citations located and those excluded, including justification | Table 1. |
| 14 | Method of addressing articles published in languages other than English | There were no year and language restrictions. |
| 15 | Method of handling abstracts and unpublished studies | We did not consider unpublished studies to be included in the analysis. |
| 16 | Description of any contact with authors | None. |
| 17 | Description of relevance or appropriateness of studies assembled for assessing the hypothesis to be tested | Detailed inclusion and exclusion criteria were described in “Eligibility criteria” of “Methods” section. |
| 18 | Rationale for the selection and coding of data (*e.g.*, sound clinical principles or convenience) | Selection of the studies was based on the inclusion and exclusion criteria. |
| 19 | Documentation of how data were classified and coded (*e.g.*, multiple raters, blinding and interrater reliability) | Mentioned in the “Data extraction” section. |
| 20 | Assessment of confounding (e.g., comparability of cases and controls in studies where appropriate) | One subgroup analysis was carried out (Fig. 3). |
| 21 | Assessment of study quality, including blinding of quality assessors, stratification or regression on possible predictors of study results | Quality assessment of each of the included studies was evaluated by MAI, SK, SSA, AHMSUP and TH based on a modified version (nine-star scoring system) of the Newcastle-Ottawa Scale (NOS) for case-control studies. |
| 22 | Assessment of heterogeneity | To assess the heterogeneity (*I²*) of the included studies, Tau-squared test was used where *I²* assessed the quantity of inconsistency across the studies (*p*<0.10 was considered as significant). A value of *I²* close to zero indicates homogeneity, whereas, the following ranges of *I²* were used to interpret heterogeneity: low heterogeneity if *I²*=25-50%, moderate heterogeneity if *I²*=51-75% and substantial heterogeneity if *I²*>75%.^14^ |
| 23 | Description of statistical methods (*e.g.*, complete description of fixed or random effects models, justification of whether the chosen models account for predictors of study results, dose-response models, or cumulative meta-analysis) in sufficient detail to be replicated | Described in the “Methods” section. |
| 24 | Provision of appropriate tables and graphics | We included one PRISMA flow diagram (Fig. 1), one main forest plot (Fig. 2), one forest plot for subgroup analysis (Fig. 3), one Labbe plot (Fig. 4), three forest plots for sensitivity analyses (Fig. 5), one contour-enhanced funnel plot (Fig. 6), one trim and fill funnel plot (Appendix C), one major characteristics table (Table 1) and one risk of bias assessment table (Table 2). |
| 25 | Graphic summarizing individual study estimates and overall estimate | Main forest plot (Fig. 2). |
| 26 | Table giving descriptive information for each study included | Major characteristics table (Table 1). |
| 27 | Results of sensitivity testing (*e.g.*, subgroup analysis) | Three forest plots for subgroup analyses (Fig. 3) and three forest plots for sensitivity analyses (Fig. 5). |
| 28 | Indication of statistical uncertainty of findings | 95% confidence intervals were presented with all summary estimates, *I*^2^ values and results of subgroup and sensitivity analyses. |
| 29 | Quantitative assessment of bias (*e.g.*, publication bias) | Funnel plots (Fig. 6 and Appendix C). |
| 30 | Justification for exclusion (*e.g.*, exclusion of non-English language citations) | None. |
| 31 | Assessment of quality of included studies | Risk of bias assessment table (Table 2). |
| 32 | Consideration of alternative explanations for observed results | Discussed in the “Discussion” section. |
| 33 | Generalization of the conclusions (i.e., appropriate for the data presented and within the domain of the literature review) | Discussed in the “Discussion” section. |
| 34 | Guidelines for future research | Discussed in the “Discussion” section. |
| 35 | Disclosure of funding source | None to declare. |
