# Supplementary material for: Prevalence of antiphospholipid antibodies in Behçet's disease: A systematic review and meta-analysis
Source: PLoS One. 2020 Jan 13;15(1):e0227836. doi: 10.1371/journal.pone.0227836 (PMC6957187; doi:10.1371/journal.pone.0227836)
Supplement: S3 Table — (DOCX) [file pone.0227836.s003.docx]

*S3 Table. Search strategies employed for PubMed, Web of Science, Embase, Scopus and ScienceDirect electronic databases.*

| **PubMed** |
| --- |
| ((Behçet[Title/Abstract]) **OR** Behcet[Title/Abstract]) **AND** (((((((((((((((((((((((((antiphospholipid[Title/Abstract]) **OR** anti-phospholipid[Title/Abstract]) **OR** phospholipid[Title/Abstract]) **OR** autoantibodies[Title/Abstract]) **OR** auto-antibodies[Title/Abstract]) **OR** cardiolipin[Title/Abstract]) **OR** anticardiolipin[Title/Abstract]) **OR** lupus anticoagulant[Title/Abstract]) **OR** annexin[Title/Abstract]) **OR** prothrombin[Title/Abstract]) **OR** phosphatidylserine[Title/Abstract]) **OR** phosphatidylinositol[Title/Abstract]) **OR** phosphatidylethanolamine[Title/Abstract]) **OR** antiphosphatidylcholine[Title/Abstract]) **OR** Anti-phosphatidylcholine[Title/Abstract]) **OR** beta 2 glycoprotein[Title/Abstract]) **OR** beta2 glycoprotein[Title/Abstract]) **OR** beta2-glycoprotein[Title/Abstract]) **OR** beta-2-glycoprotein[Title/Abstract]) **OR** β2GP[Title/Abstract]) **OR** β2-GP[Title/Abstract]) **OR** β-2-GP[Title/Abstract]) **OR** β2glycoprotein[Title/Abstract]) **OR** β2 glycoprotein[Title/Abstract]) **OR** β-2-glycoprotein[Title/Abstract]) |
| **Web of Science** |
| (TI=Behçet **OR** TI=Behcet) **AND** (TI=antiphospholipid **OR** TI=anti-phospholipid **OR** TI=phospholipid **OR** TI=autoantibodies **OR** TI=auto-antibodies **OR** TI=anticardiolipin **OR** TI=lupus anticoagulant **OR** TI=beta2 glycoprotein **OR** TI=beta2-glycoprotein **OR** TI=beta-2-glycoprotein **OR** TI=β2GP **OR** TI=β2-GP **OR** TI=β-2-GP **OR** TI=β2glycoprotein **OR** TI=β2-glycoprotein **OR** TI=β-2-glycoprotein **OR** TI=annexin **OR** TI=prothrombin **OR** TI=phosphatidylserine **OR** TI=phosphatidylinositol **OR** TI=phosphatidylethanolamine **OR** TI=cardiolipin) |
| **Embase** |
| ('Behçet':ti **OR** 'Behcet':ti) **AND** ('antiphospholipid':ti **OR** 'anti-phospholipid':ti **OR** 'phospholipid':ti **OR** 'autoantibodies':ti **OR** 'auto-antibodies':ti **OR** 'anticardiolipin':ti **OR** 'lupus anticoagulant':ti **OR** 'beta2glycoprotein':ti **OR** 'beta2-glycoprotein':ti **OR** 'beta-2-glycoprotein':ti **OR** 'β2gp':ti **OR** 'β2-gp':ti **OR** 'β-2-gp':ti **OR** 'β2glycoprotein':ti **OR** 'β2-glycoprotein':ti **OR** 'β-2-glycoprotein':ti **OR** 'annexin':ti **OR** 'prothrombin':ti **OR** 'phosphatidylserine':ti **OR** 'phosphatidylinositol':ti **OR** 'phosphatidylethanolamine':ti **OR** 'cardiolipin':ti) |
| **Scopus** |
| TITLE("Behçet" **OR** " Behcet") **AND** TITLE("antiphospholipid" **OR** "anti-phospholipid" **OR** "phospholipid" **OR** "autoantibodies" **OR** "auto-antibodies" **OR** "anticardiolipin" **OR** “lupus anticoagulant” **OR** "beta2 glycoprotein" **OR** "beta2-glycoprotein" **OR** "beta-2-glycoprotein" **OR** "β2GP" **OR** "β2-GP" **OR** "β-2-GP" **OR** "β2glycoprotein" **OR** "β2-glycoprotein" **OR** "β-2-glycoprotein" **OR** "annexin" **OR** "prothrombin" **OR** "phosphatidylserine" **OR** "phosphatidylinositol" **OR** "phosphatidylethanolamine" **OR** "cardiolipin") |
| **ScienceDirect** |
| Title-Abstr-key("Behçet" **OR** "Behcet") **AND** Title-Abstr-key("antiphospholipid" **OR** "anti-phospholipid" **OR** "phospholipid" **OR** "autoantibodies" **OR** "auto-antibodies" **OR** "anticardiolipin" **OR** “lupus anticoagulant” **OR** "beta2 glycoprotein" **OR** "beta2-glycoprotein" **OR** "beta-2-glycoprotein" **OR** "β2GP" **OR** "β2-GP" **OR** "β-2-GP" **OR** "β2glycoprotein" **OR** "β2-glycoprotein" **OR** "β-2-glycoprotein" **OR** "annexin" **OR** "prothrombin" **OR** "phosphatidylserine" **OR** "phosphatidylinositol" **OR** "phosphatidylethanolamine" **OR** "cardiolipin") |
