# Supplementary material for: Prevalence of antiphospholipid antibodies in Behçet's disease: A systematic review and meta-analysis
Source: PLoS One. 2020 Jan 13;15(1):e0227836. doi: 10.1371/journal.pone.0227836 (PMC6957187; doi:10.1371/journal.pone.0227836)
Supplement: S2 Fig — Studies within the limits are interpreted as homogeneous. (DOCX) [file pone.0227836.s005.docx]

*S2 Fig. Galbraith plot representing the possible sources of heterogeneity. Studies within the limits are interpreted as homogeneous.*
